# Tetracycline Removal through the Synergy of Catalysis and Photocatalysis by Novel NaYF_4_:Yb,Tm@TiO_2_-Acetylacetone Hybrid Core-Shell Structures

**DOI:** 10.3390/ijms24119441

**Published:** 2023-05-29

**Authors:** Lidija Mančić, Lucas A. Almeida, Tamires M. Machado, Jessica Gil-Londoño, Ivana Dinić, Miloš Tomić, Smilja Marković, Paula Jardim, Bojan A. Marinkovic

**Affiliations:** 1 Institute of Technical Sciences of SASA, 11000 Belgrade, Serbia; ivana.dinic@itn.sanu.ac.rs (I.D.); milos.tomic@itn.sanu.ac.rs (M.T.); smilja.markovic@itn.sanu.ac.rs (S.M.); 2 Department of Chemical and Materials Engineering, Pontifical Catholic University of Rio de Janeiro (PUC-Rio), Rio de Janeiro 22453-900, Brazil; 3 Department of Metallurgical and Materials Engineering, Federal University of Rio de Janeiro, Rio de Janeiro 21941-853, Brazil

**Keywords:** visible light photocatalysis, antibiotic removal, core-shell, up-conversion, ligand-to-metal charge transfer, FRET

## Abstract

Novel hybrid core-shell structures, in which up-converting (UC) NaYF_4_:Yb,Tm core converts near-infrared (NIR) to visible (Vis) light via multiphoton up-conversion processes, while anatase TiO_2_-acetylacetonate (TiO_2_-Acac) shell ensures absorption of the Vis light through direct injection of excited electrons from the highest-occupied-molecular-orbital (HOMO) of Acac into the TiO_2_ conduction band (CB), were successfully synthesized by a two-step wet chemical route. Synthesized NaYF_4_:Yb,Tm@TiO_2_-Acac powders were characterized by X-ray powder diffraction, thermogravimetric analysis, scanning and transmission electron microscopy, diffuse-reflectance spectroscopy, Fourier transform infrared spectroscopy, and photoluminescence emission measurement. Tetracycline, as a model drug, was used to investigate the photocatalytic efficiencies of the core-shell structures under irradiation of reduced power Vis and NIR spectra. It was shown that the removal of tetracycline is accompanied by the formation of intermediates, which formed immediately after bringing the drug into contact with the novel hybrid core-shell structures. As a result, ~80% of tetracycline is removed from the solution after 6 h.

## 1. Introduction

The application of photocatalysis for pollution treatment is an environmentally friendly approach based on the power of semiconductors to generate intrinsic reactive oxygen species (ROS) under sunlight. Among various photocatalysts reported so far, anatase (TiO_2_), although significantly active only under ultraviolet (UV) light, is undoubtedly the most widely used semiconductor for the degradation of pollutants, due to its low cost, extraordinary chemical stability, and biocompatible features [1]. Much effort has been devoted to extending TiO_2_ absorption to visible light through many different approaches, such as noble metal doping, creation of defects, and heterojunction formation, to mention some of them, but it was found that the overall photocatalytic capability can be affected by the recombination of photogenerated charge carriers [2,3]. Recently, a new approach of creating ligand-to-metal charge transfer (LMCT) complex is recognized to be a promising path for overcoming this problem [4]. Alternatively, the maximization of sunlight harvesting is shown to be possible through the creation of composites, hybrid, or core-shell structures, thanks to the synergy of functional diversities that come from comprising compounds [5,6,7]. In the past decade, several up-converting materials were explored as core in TiO_2_-based core-shell structures, due to their unique capability of converting NIR into UV-Vis light [8,9,10]. The Yb,Tm doped β-NaYF_4_ phase is shown to be the most appropriate choice for that, since Tm^3+^ ions UV emission matches well with the absorption band of TiO_2_ [11]. Mostly, studies have shown that NaYF_4_Yb,Tm@TiO_2_ enables efficient degradation of dyes under NIR light [12,13,14,15]. There are only a few reports on sunlight-driven photocatalytic efficiency toward the degradation of other pollutants, such as microcystin [11] and ciprofloxacin [16]. Beside the radiation-reabsorption process, Förster resonance energy transfer (FRET) is recognized as an additional energy migration process which occurs between core and shell [12]. However, although the up-conversion process in NaYF_4_:Yb,Tm core provokes the increase of visible light photons in TiO_2_ shell, the harvesting of the visible light is not achieved, due to low absorption of neat TiO_2_ in this region.

Bearing the above in mind, the current work presents, for the first time as the authors are aware, catalytic and photocatalytic activity, towards tetracycline, of the novel core-shell structures, in which TiO_2_-based shell is sensitive to the Vis light. We already showed that the formation of LMCT in TiO_2_-Acac nanoparticles effectively shifts TiO_2_ absorption to Vis range, boosting its photocatalytic efficiency toward different pollutants [17,18,19]. In this work, an additional light-harvesting feature was achieved by the NIR-to-Vis conversion capability of β-NaYF_4_:Yb,Tm phase. As expected, the synergy of a broad spectrum absorption in calcined NaYF_4_:Yb,Tm@TiO_2_-Acac core-shell structure improved tetracycline degradation under joint irradiation of reduced power Vis and NIR spectra. In addition, it was found that catalysis precedes to photocatalysis, contributing to the overall tetracycline removal.

It Is relevant to note that tetracycline is one of the most widely used antibiotics in humans and in domestic animals, so developing effective sunlight-driven photocatalysts for its removal from wastewater represents a great challenge. To address this issue, besides TiO_2_-Acac and TiO_2_-glutaric acid charge transfer complexes [18,19,20], Co-TiO_2_ mesoporous structures [21], CdS/TiO_2_, Cu_2_O/TiO_2_, and MoS_2_/TiO_2_ composites [22,23,24], magnetic graphene oxide loaded TiO_2_:Ce [25], and TiO_2_/Fe_2_O_3_ heterojunction [26] were tested up to now, but there is no report about photocatalytic performance of the UC@TiO_2_-based core-shell structures.

## 2. Results

**Figure 1:**
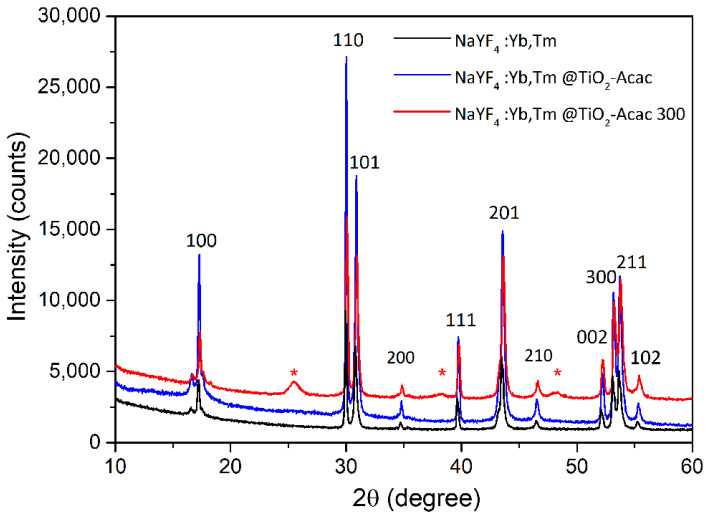
XRPD patterns of NaYF_4_:Yb,Tm, NaYF_4_:Yb,Tm@TiO_2_-Acac, and NaYF_4_:Yb,Tm@TiO_2_-Acac 300 samples. The diffraction lines of anatase TiO_2_ are marked with *. The diffraction lines of β-NaYF_4_ are indexed accordingly.

Based on the X-ray powder diffraction (XRPD) patterns presented in Figure 1, NaYF_4_:Yb,Tm powder obtained through the hydrothermal processing presents a hexagonal β-NaYF_4_ structure (JCPDS 28-1192) and is monophasic. The absence of diffraction lines due to secondary phases suggests the effective incorporation of dopants in the hexagonal crystal lattice. Coating of β-NaYF_4_:Yb,Tm particles with TiO_2_-Acac did not cause changes in the XRPD pattern of NaYF_4_:Yb,Tm@TiO_2_-Acac. The absence of additional diffraction lines for the NaYF_4_:Yb,Tm@TiO_2_-Acac sample belonging to TiO_2_ could be an indication of an amorphous or nanocrystalline TiO_2_-Acac shell formation. With the subsequent calcination at 300 °C, the three strongest TiO_2_ anatase diffraction lines appeared in the pattern of the NaYF_4_:Yb,Tm@TiO_2_-Acac 300 sample. These findings corroborate well with the previous reports on low-crystalline and/or amorphous TiO_2_-Acac xerogel formation using a similar sol-gel processing route [27,28], and are additionally supported by selected area electron diffraction (SAED) analysis (*vide infra*).

**Figure 2:**
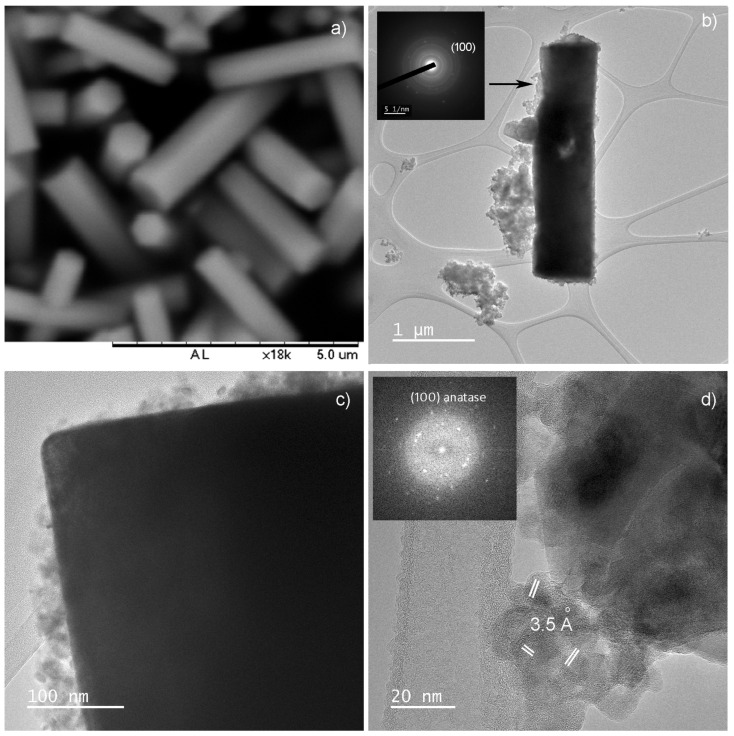
SEM of NaYF_4_:Yb,Tm hexagonal prisms (**a**); TEM/SAED of NaYF_4_:Yb,Tm@TiO_2_-Acac core-shell structure (**b**); TEM of NaYF_4_:Yb,Tm@TiO_2_-Acac 300 core-shell structure (**c**); and HRTEM/FFT of NaYF_4_:Yb,Tm@TiO_2_-Acac 300 of core-shell structure (**d**).

**Figure 3:**
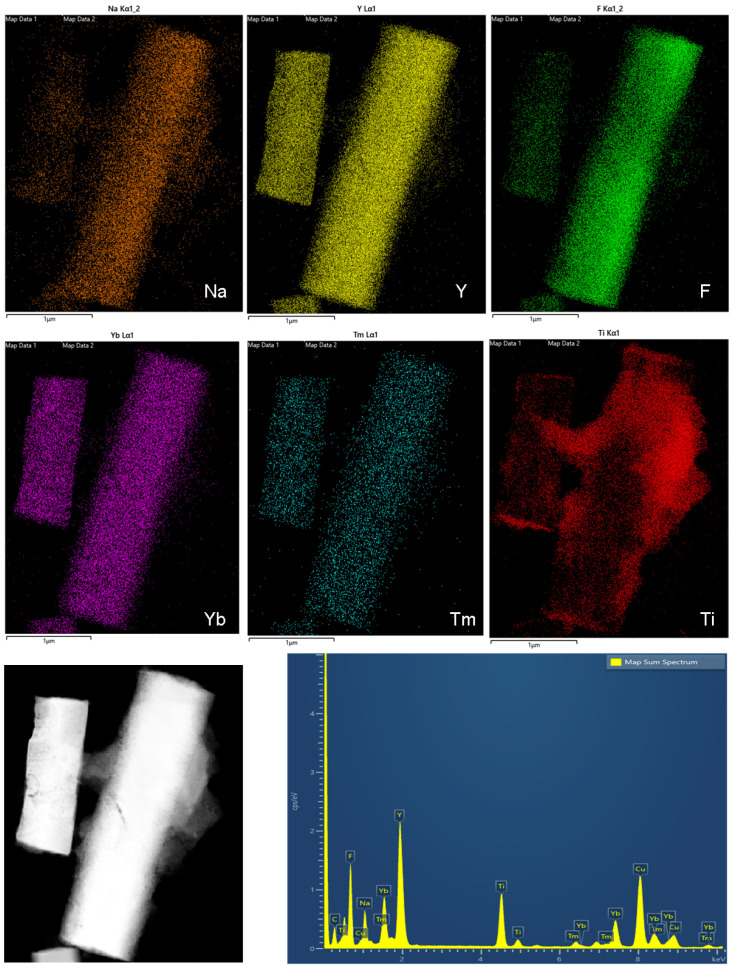
Element mapping and STEM/EDXS of the NaYF_4_:Yb,Tm@TiO_2_-Acac 300 core-shell structure.

The morphology of NaYF_4_:Yb,Tm particles before and after coating was revealed by scanning and transmission electron microscopy (SEM and TEM), Figure 2 and Figure S1. The as-prepared particles adopt a shape of well-defined hexagonal prisms with a longer edge of about 3 μm, along the c-axis, and the shorter ones less than 500 nm (Figure 2a). As it is reported previously [29,30], observed morphology is the consequence of the inherent anisotropic growth of hexagonal NaYF_4_ phase along [0001] direction. After coating and calcination, a rugged TiO_2_-Acac layer, with a thickness up to several tens of nanometers, was formed on the NaYF_4_:Yb,Tm particles, forming a core-shell structure, as illustrated in the TEM images of Figure 2b,c and Figure S2b. A SAED inset in Figure 2b confirms nanocrystalline anatase in the NaYF_4_:Yb,Tm@TiO_2_-Acac sample, while the high resolution transmission microscopy and fast Fourier transform analysis (HRTEM/FFT), Figure 2d, reveal the presence of anatase (100) crystallographic planes in the NaYF_4_:Yb,Tm@TiO_2_-Acac 300 structure. As it is notable from Figure S2a, TiO_2_-Acac shell in NaYF_4:_Yb,Tm@TiO_2_-Acac presents a porous structure, and is loosely, and not uniformly, coupled to the NaYF_4_:Yb,Tm core. After calcination, a more compact shell of TiO_2_-Acac nanoclusters was formed (Figure 2c and Figure S2b), ensuring the existence of a high number of contact points between core and shell. The high chemical purity and compositional homogeneity of core-shell structures were confirmed by scanning transmission electron microscopy coupled energy dispersive X-ray spectroscopy (STEM/EDXS). The distribution of titanium over a single NaYF_4_:Yb,Tm particle is presented in Figure 3.

**Figure 4:**
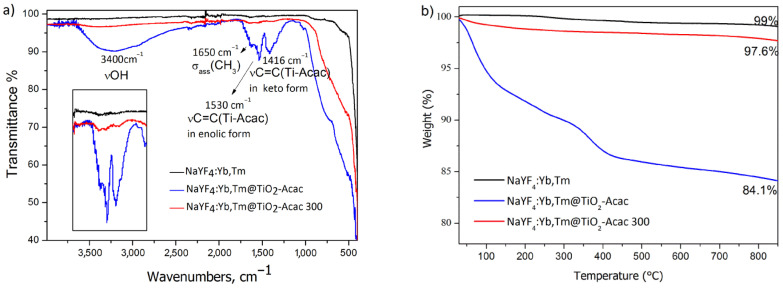
FTIR spectra (**a**) and TG curves (**b**) of NaYF_4_:Yb,Tm particles, NaYF_4_:Yb,Tm@TiO_2_-Acac and NaYF_4_:Yb,Tm@TiO_2_-Acac 300 core-shell structures.

The Fourier transform infrared (FTIR) spectrum of NaYF_4_:Yb,Tm@TiO_2_-Acac, presented in Figure 4a, implicates the preservation of Acac, interacting with anatase onto the surface of core-shell structures. Strong bends at 1416, 1530, and 1650 cm^−1^ are attributed to the methyl asymmetric bending, as well as the covalent and dipole chemical reaction of acetylacetonate with Ti^4+^ ion [17]. As shown in our previous study [17], calcination of TiO_2_-Acac CT at 300 °C leads to decrease of carbon, i.e., Acac content, so these bands present only faint intensities in the spectrum of the NaYF_4_:Yb,Tm@TiO_2_-Acac 300 structure. The presence of the adsorbed water in NaYF_4_:Yb,Tm@TiO_2_-Acac is identified through the band of OH stretching vibration, situated at 3400 cm^−1^.

The results obtained through thermogravimetric analysis (TGA), Figure 4b, corroborate well with these findings. A total mass loss of 16 wt%, recorded during heating of NaYF_4_:Yb,Tm@TiO_2_-Acac, is owing to the TiO_2_-Acac shell and occurs in three stages, in accordance to [17,18]: (a) moisture loss (up to 150 °C), (b) volatilization of acetyl ions, acetone, and CO_2_ (up to 300 °C), and (c) release of CO_2_ at higher temperatures. On the other hand, negligible mass loss was detected during heating of neat NaYF_4_:Yb,Tm, while, for the NaYF_4_:Yb,Tm@TiO_2_-Acac 300 sample, a low mass loss (<3%) was recorded, corresponding to the Acac, remaining after the calcination stage at 300 °C.

**Figure 5:**
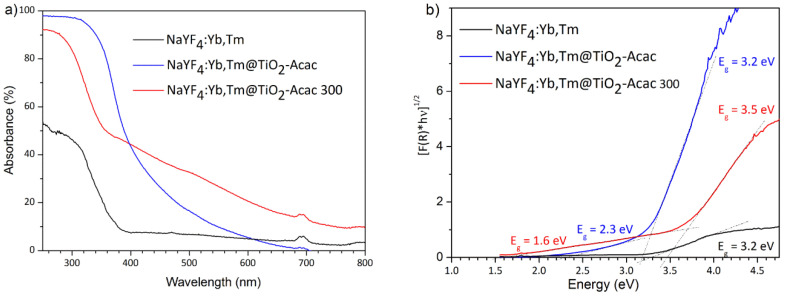
DRS (**a**) and Kubelka-Munk plots (**b**) of NaYF_4_:Yb,Tm, NaYF_4_:Yb,Tm@TiO_2_-Acac and NaYF_4_:Yb,Tm@TiO_2_-Acac 300 core-shell structures.

The optical properties of studied samples were investigated by diffuse reflectance spectroscopy (DRS). As shown in Figure 5a, NaYF_4_:Yb,Tm@TiO_2_-Acac and NaYF_4_:Yb,Tm@TiO_2_-Acac 300 absorbed significantly more UV and Vis light than the neat NaYF_4_:Yb,Tm. A large absorption tail within the visible spectral range with the apparent band-gaps of 2.3 and 1.6 eV for NaYF_4_:Yb,Tm@TiO_2_-Acac and NaYF_4_:Yb,Tm@TiO_2_-Acac 300, respectively, were evaluated from the Kubelka-Munk plots (Figure 5b). The visible spectrum tail recorded for NaYF_4_:Yb,Tm@TiO_2_-Acac (Figure 5) may be fully ascribed to the ligand-to-metal charge transfer phenomenon between TiO_2_-Acac, where the electrons from the highest occupied molecular orbital (HOMO) of acetylacetone are directly injected into the conduction band (CB) of TiO_2_ [17]. However, even more intense absorption within the visible spectrum of NaYF_4_:Yb,Tm@TiO_2_-Acac 300 can be additionally attributed to the extrinsic oxygen vacancies generated in the anatase shell layer through oxidation decomposition of Acac [18,19]. The same feature was recently reported for another charge transfer complex, namely, TiO_2_-glutaric acid, after calcination at 270 °C [20]. The characteristic absorption feature of the neat NaYF_4_:Yb,Tm is preserved in core-shell structures, since a small peak at ∼690 nm, attributed to ^3^H_6_→^3^F_2,3_ Tm^3+^ transitions, is still visible.

**Figure 6:**
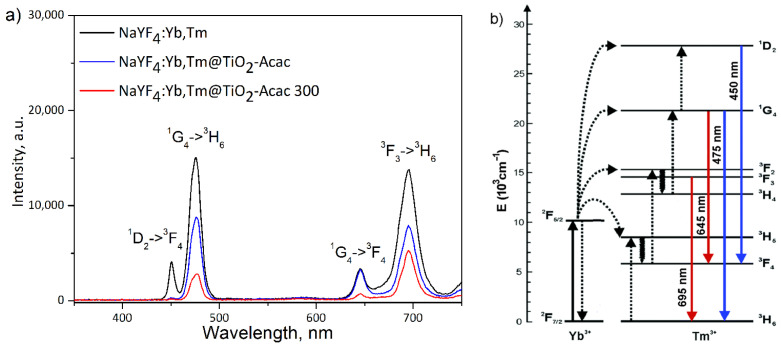
UC emission spectra of neat NaYF_4_:Yb,Tm, NaYF_4_:Yb,Tm@TiO_2_-Acac, and NaYF_4_:Yb,Tm@TiO_2_-Acac 300 core-shell structures (**a**) and electron energy level diagram of Yb^3+^ and Tm^3+^ ions (**b**). Solid, dotted, and wavy arrows represent photon absorption or emission, energy transfer, and relaxation processes, respectively.

Figure 6a shows the UC emission spectra of β-NaYF_4_:Yb,Tm, before and after coating, under 976 nm excitation. The visible emissions from ^1^D_2_→^3^F_4_, ^1^G_4_→^3^H_6_, and ^1^G_4_→^3^F_4_, and ^3^F_2,3_→^3^H_6_ transitions of Tm^3+^ ions were detected at 450, 475, 645, and 695 nm, respectively. As shown in Figure 6b, the population of the excited states in Tm^3+^ ions is due to energy transfer from the Yb^3+^ sensitizer. Initially, Yb^3+^ ions were excited through absorption of the incident photons from the ^2^F_7/2_ ground state to the ^2^F_5/2_ excited state. Then, Tm^3+^ ions in the ^3^H_6_ ground state are excited to ^3^H_5_ by the neighboring excited Yb^3+^ ions through the process: ^3^H_6_(Tm^3+^)+^2^F_5/2_(Yb^3+^)→^3^H_5_(Tm^3+^)+^2^F_7/2_(Yb^3+^). Subsequently, Tm^3+^ ions transit to the ^3^F_4_ state by nonradiative relaxation, and with the supplementary energy transfer from Yb^3+^ populate ^3^F_2,3_ states. With ^3^F_3_ relaxation to the ground level, red emission centered at 695 nm occurs. Moreover, Tm^3+^ ions in ^3^F_2_, which relax to ^3^H_4_ state by nonradiative relaxation, are further excited to ^1^G_4_ state by following the energy transfer process: ^3^H_4_(Tm^3+^)+^2^F_5/2_(Yb^3+^)→^1^G_4_(Tm^3+^)+^2^F_7/2_(Yb^3+^). With the consequent ^1^G_4_→^3^H_6_ and ^1^G_4_→^3^F_4_ relaxation, blue emission at 475 nm and red emission at 645 nm appeared, respectively. Ultimately, for the population of the ^1^D_2_ state, one extra energy transfer is needed to enable blue emission at 475 nm. Therefore, as it is shown in previous studies [31,32], appearance of the UC emission in the visible part of spectra is a result of two- and three-photon processes, both ensured by the energy transfer from Yb^3+^ to Tm^3+^. As it is notable from Figure 6a, the blue and red emission decrease unequally with the coating and subsequent calcination. While the intensity ratio of blue emission decreased twice after coating of NaYF_4_:Yb,Tm particles, and three times with the subsequent calcination, red emission reduces for about 1.8 times after each of these processes. As a result, the blue-to-red intensity ratio decreased from 0.66 in NaYF_4_:Yb,Tm to 0.58, and 0.36 in NaYF_4_:Yb,Tm@TiO_2_-Acac and NaYF_4_:Yb,Tm@TiO_2_-Acac 300 core-shell structures, respectively. This confirms that the TiO_2_-Acac shell is capable of absorbing a part of visible light produced through excitation of the NaYF_4_:Yb,Tm core by 976 nm, since absorption bands of core-shell structures, Figure 5, overlap particularly well with the UC blue emission. As expected, absorption is more powerful in the NaYF_4_:Yb,Tm@TiO_2_-Acac 300 core-shell structure than in NaYF_4_:Yb,Tm@TiO_2_-Acac, because of the better shell connection to the UC core (Figure 2c and Figure S2b).

**Figure 7:**
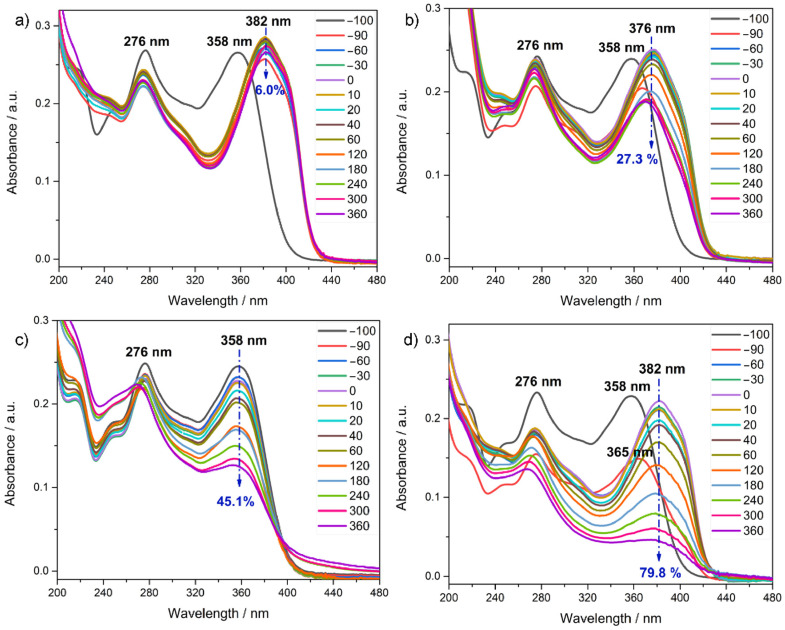
UV-Vis absorption spectra for the aliquots collected during tetracycline degradation in the presence of the different photocatalysts: (**a**) neat NaYF_4_:Yb,Tm, (**b**) NaYF_4_:Yb,Tm@TiO_2_-Acac, (**c**) TiO_2_-Acac 300, and (**d**) NaYF_4_:Yb,Tm@TiO_2_-Acac 300. The percentages correspond to the photocatalytic degradation.

The photocatalytic efficiency of core-shell structures was evaluated based on tetracycline degradation during the irradiation period of 6 h. Figure 7 shows the UV-Vis absorption spectra of aliquots collected during tetracycline degradation in the presence of different photocatalysts. The pure tetracycline (previous to the addition of the photocatalyst) exhibits, inherently, two strong absorption bands, centered at around 276 nm and 358 nm (identified as −100 curve in Figure 7). The absorption band centered at 276 nm is attributed to the ring A and dimethylamino group, while the second band (around 358 nm) corresponds to the aromatic rings B, C, and D, and the developing chromophores, as represented in [33,34]. As can be noted from Figure 7, the addition of neat NaYF_4_:Yb,Tm and core-shell structures into tetracycline solution under darkness induced a red shift of the band centered at 358 nm. Neat NaYF_4_:Yb,Tm exhibited a red shift from 358 nm to 382 nm (Figure 7a), while NaYF_4_:Yb,Tm@TiO_2_-Acac showed a shift toward 376 nm (Figure 7b). Moreover, NaYF_4_:Yb,Tm@TiO_2_-Acac 300 displayed an initial shift from 358 nm to 365 nm after 10 min of contact between the pollutant and photocatalyst under darkness, and another one after 40 min, from 365 nm to 382 nm (Figure 7d). As it will be discussed later, these shifts may indicate some catalytic activity of the NaYF_4_:Yb,Tm and, consequently, the formation of intermediate products. Contrary to that, such a catalytic activity is not noted for TiO_2_-Acac 300, Figure 7c. The adsorption-desorption equilibrium in darkness (1 h) was followed by the photocatalytic degradation of tetracycline (for the case of TiO_2_-Acac 300, Figure 7c), and the degradation of intermediate products (for neat NaYF_4_:Yb,Tm and core-shell structures, Figure 7a,b,d) during the mentioned irradiation period.

**Figure 8:**
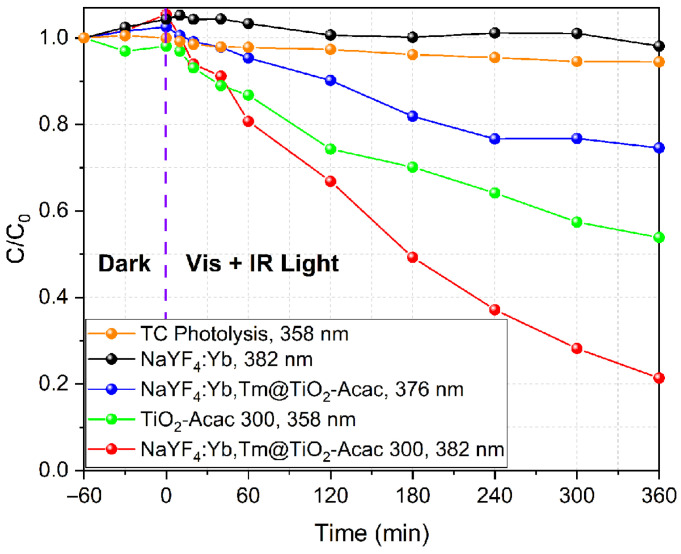
Change in the tetracycline and intermediate compound concentrations during testing period in darkness (adsorption-desorption equilibrium) and under Vis+IR irradiation.

Figure 8 shows the change in the tetracycline and intermediate product concentrations during the testing period. As demonstrated by Figure 8, photolysis showed negligible tetracycline degradation, and neat NaYF_4_:Yb,Tm remained photocatalytically inactive under Vis+IR irradiation, as well. On the other hand, the TiO_2_-Acac 300 and NaYF_4_:Yb,Tm@TiO_2_-Acac core-shell structures showed different photocatalytic degradation of tetracycline and of intermediates, respectively. With irradiation, the photocatalytic degradation of intermediates reached about 27.3 and 79.8% after 6 h in the presence of 200 mg L^−1^ of NaYF_4_:Yb,Tm@TiO_2_-Acac and NaYF_4_:Yb,Tm@TiO_2_-Acac 300 core-shell structures, respectively (Figure 7 and Figure 8). On the other hand, in the presence of 25 mg L^−1^ of TiO_2_-Acac 300 nanoparticles (equal to the amount of shell nanomaterial present in the core-shell structure), the degradation of 45.1% of tetracycline was achieved during the same period.

As expected, the visible light emission produced by the NaYF_4_:Yb,Tm core, in addition to visible lamp irradiation, increased the photocatalytic activity of NaYF_4_:Yb,Tm@TiO_2_-Acac 300 in comparison to TiO_2_-Acac 300 (Figure 8). The existence of a higher number of contact points between core and shell, and the presence of much stronger bonds between Acac and TiO_2_ in the NaYF_4_:Yb,Tm@TiO_2_-Acac 300, compared to the NaYF_4_:Yb,Tm@TiO_2_-Acac core-shell structure (Figure 2 and Figure S2), improved the photocatalytic performance in the degradation of tetracycline intermediates. Additionally, the enhanced tetracycline photocatalytic degradation by NaYF_4_:Yb,Tm@TiO_2_-Acac 300, compared to TiO_2_-Acac 300, can be an indicator of the synergy of catalytic and photocatalytic processes in the novel hybrid NaYF_4_:Yb,Tm@TiO_2_-Acac 300 core-shell structure.

## 3. Discussion

**Figure 9:**
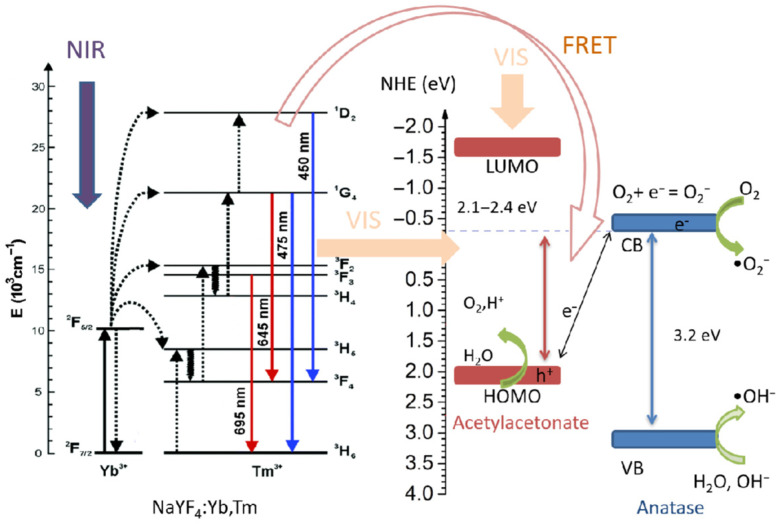
Schematic representation of the electronic bands in the NaYF_4_@TiO_2_–Acac core-shell structure, and possible ways for increased generation of reactive species.

Novel hybrid core-shell NaYF_4_:Yb,Tm@TiO_2_-Acac structures were employed as a catalyst and photocatalyst for tetracycline removal. The proposed mechanism of the photocatalysis is illustrated in Figure 9. Under irradiation, the core absorbed NIR light, while the shell absorbed Vis light. Due to the successful NIR-to-Vis multiphoton up-conversion, radiation-reabsorption and FRET processes are enabled in core-shell structures. To confirm the energy transfer from core to shell, time-resolved fluorescence of Tm^3+^ was measured for NaYF_4_:Yb,Tm hexagonal prisms and core-shell structures. In accordance with Figure S3, the best fits of decays were obtained by employing the bi-exponential functions. Values of τ_1_:111 µs τ_2_:388 µs (NaYF_4_:Yb,Tm), τ_1_:103 µs τ_2_:329 µs (NaYF_4_:Yb,Tm@TiO_2_-Acac), and τ_1_:104 µs τ_2_:333 µs (NaYF_4_:Yb,Tm@TiO_2_-Acac 300) confirmed a shortening of the decay time after coating, due to formation of new energy transfer channels from the excited states of Tm^3+^ to TiO_2_-Acac. As a result, an increased number of reactive oxygen species are generated on the TiO_2_-Acac shell. As shown in some previous studies through the correlation between EPR-spin trapping and tetracycline photodegradation using scavengers [18,19], tetracycline degradation over TiO_2_-Acac occurs via interactions with electronic holes and, principally, superoxide radicals. On the other hand, catalytic activity of the NaYF_4_:Yb,Tm-based structures was corroborated through shifting of the tetracycline absorption band, originally situated at 358 nm. Although there is no literature data, as the authors are aware, related to the catalytic property of neat NaYF_4_:Yb,Tm or NaYF_4_:Yb,Tm@TiO_2_ core-shell structures toward liquid or gas pollutants, the formation of tetracycline intermediates in darkness, followed by the band shifting, has been already reported for ZnO Nanorods/K-Doped Exfoliated g-C_3_N_4_ nanosheets [35], modified bismuth tungstate nanoparticles [36], and polyoxometalates/polymer composites [37]. A red shifting of the absorption band centered at 358 nm is also referred to the feature of tetracycline to chelate with metal ions [33,34]. The tendency of tetracycline to complex with divalent and trivalent metal ions is due to the presence of electron-rich ketone, carboxyl, amino, and hydroxyl groups in its structure [38,39]. Yu et al. [34] reported that the photodegradation of tetracycline under simulated sunlight, in the presence of La_2-x_Sr_x_NiMnO_6_, is enhanced by its chelation with Sr^2+^ in darkness. Therefore, the red shifting of the absorption band from 358 nm to 390 nm may be assigned to the conformational transformation in tetracycline to form a complex [34]. Further research should be undertaken to deduce the exact mechanism which induces band shifting in tetracycline absorption spectra in the presence of NaYF_4_:Yb,Tm-based structures.

## 4. Materials and Methods

### 4.1. Materials

All reagents were purchased from Sigma-Aldrich (St. Louis, MO, USA) and used as obtained. Rare earth (RE) nitrates [Y(NO_3_)_3_**•**6H_2_O, Yb(NO_3_)_3_**•**5H_2_O and Tm(NO_3_)_3_**•**5H_2_O], NaF (≥99%), ethanol (≥99%), and Ethylenediaminetetraacetic acid (EDTA, 99.4–100.6%) were used for NaYF_4_:Yb,Tm particle synthesis. Titanium isopropoxide (TTIP, 97%), acetylacetone (Acac, ≥99%), ethanol (≥99.8%), and nitric acid (65%) were used in the process of NaYF_4_:Yb,Tm particle coating. Tetracycline (TC, ≥98%) was used as an aqueous solution with concentration of 10 mg L^−1^ for photocatalysis test. 

### 4.2. Synthesis of β-NaYF_4_:Yb,Tm Particles (Core)

Up-converting particles with a nominal composition NaY_0.78_Yb_0.2_Tm_0.02_F_4_ were hydrothermally synthesized in the presence of EDTA using common rare earth (RE) nitrate precursor (0.01 M) and NaF (RE:EDTA ratio of 1 and RE:F ratio of 14), in accordance with the previously established route [29]. The ethanol-water mixture (2:1) was used as a solvent. The synthesis was performed in PTFE linen Berghof autoclave, BR-300 (Berghof Products + Instruments GmbH, Eningen, Germany) at 200 °C (2 h), under stirring. The as-obtained particles were separated through centrifugation (7000 rpm), rinsed with deionized water, and dried at 100 °C (2 h).

### 4.3. Formation of TiO_2_-Acac Shell onto β-NaYF_4_:Yb,Tm Particles 

The previously used procedure for the synthesis of TiO_2_-Acac nanoparticles [27] was adopted here for the deposition of a 50 nm-thick TiO_2_-Acac shell over NaYF_4_:Yb,Tm particles. For this purpose, 120 mg of as-obtained NaYF_4_:Yb,Tm particles were dispersed in 1270 mL of ethanol, with the aid of stirring and ultrasonication. Then, 127 mL of Acac:Ethanol solution (1:5) was added dropwise under continual stirring. After that, 183 mL of TTIP was added under vigorous stirring for 40 min. To initiate the hydrolysis reaction of TTIP, 1098 mL of 0.015 M HNO_3_ was added under magnetic stirring, and the obtained mixture was kept under heating at 60 °C until condensation and reddish NaYF_4_:Yb,Tm@TiO_2_-Acac xerogel formation was finished. Xerogel, previously grounded in an agate mortar, was dried at 100 °C, and the core-shell particles obtained after drying were denoted as NaYF_4_:Yb,Tm@TiO_2_-Acac. A part of these was ultimately calcined at 300 °C for 2 h (NaYF_4_:Yb,Tm @TiO_2_-Acac 300). 

### 4.4. Characterization

The materials phase composition was examined by X-ray powder diffraction (XRPD) at room temperature, using a Bruker diffractometer D8 Advance (Bruker, Billerica, MA, USA) with CuKα radiation (λ = 1.5418 Å) and 2θ scanning rate of 0.02 °/s. The morphological and crystallographic particles characteristics were investigated using scanning electron microscopy (SEM, Hitachi TM3000 operating at 15 kV, Hitachi High-Technologies Corporation, Tokio, Japan) and transmission electron microscopy (TEM, Jeol JEM-2100F, Tokio, Japan). The images were acquired in conventional TEM and scanning TEM mode, coupled with energy-dispersive X-ray spectroscopy (EDXS). Thermogravimetric analysis (TGA) was performed on a Perkin-Elmer Simultaneous Thermal Analyzer STA-6000 (Perkin-Elmer, Waltham, MA, USA) under airflow (20 mL min^−1^) within the temperature range from 30 to 800 °C. The heating rate was 10 °C min^−1^. Fourier transform infrared spectroscopy (FTIR) was done on a Nicolet iS10 FT-IR Spectrometer (Thermo Scientific, Waltham, MA, USA) in the spectral range from 400 to 4000 cm^−1^. UV-Vis absorption spectra were obtained in a diffuse reflection mode using a Perkin-Elmer Lambda 650 UV/Vis spectrophotometer (Perkin-Elmer, Waltham, MA, USA). Absorption data were used as input for Kubelka-Mulk function to determine band-gap energies. The photoluminescence emission spectroscopy (PL) at room temperature was performed using a TE Cooled CCD Fluorescence spectrometer (Glacier X, BWTEK, Plainsboro, NJ, USA) and a laser diode of 976 nm. For measuring of time resolved fluorescence, a RTB2004 Rohde & Schwarz digital oscilloscope was used.

The photocatalytic degradation of tetracycline solution (10 mg L^−1^) was performed using the procedure previously described in [18]. Irradiation source comprised DULUX D/E 26 W residential fluorescent lamp with an irradiance of 0.23 W cm^−2^ (400–700 nm) and NIR LED diode SMB1N-980D of 400 mW (RoithnerLaserTechnik, Wien, Austria). The tetracycline degradation tests were performed under vigorous magnetic stirring, using 200 mg L^−1^ of the core-shell material. Comparative photocatalytic tests were completed using 25 mg L^−1^ of TiO_2_-Acac nanoparticles, since this amount corresponds to the theoretical mass of the 50 nm-thick TiO_2_-Acac shell. After ensuring the adsorption–desorption equilibrium in darkness (1 h) the lamps were turned on, and 5 mL aliquots of supernatant were acquired at designed time intervals. The supernatant was double-filtered, using a Merck Millipore filter (0.45 µm), and analyzed by a UV-Vis spectrophotometer (Agilent 8453, Santa Clara, CA, USA). The absorption band situated at 358 nm was used to determine the percentage of tetracycline photodegradation over a period of time (up to 6h) by comparing the absorption of the filtered supernatant with that of the initial tetracycline solution. For tetracycline intermediates, photodegradation was determined from their corresponding absorption bands, centered at 376 nm and at 382 nm.

The analyses of all the measurement data were performed using MS Excel (Office LTSC Standard 2021 Education). 

## 5. Conclusions

In this work, novel hybrid core-shell structures with broad-spectrum absorption were successfully synthesized by a two-step wet chemical route. The core, composed of up-converting NaYF_4_:Yb,Tm microcrystals, was prepared through the EDTA-assisted hydrothermal process, while the shell of anatase TiO_2_-Acac was formed via the sol-gel method. Owing to the fact that ^1^D_2_→^3^F_4_ and ^1^G_4_→^3^H_6_ emission of Tm^3+^ matches well with the absorption edge of TiO_2_-Acac, radiation-reabsorption and FRET processes occur in core-shell structures, improving the overall generation of reactive oxygen species and improved degradation of tetracycline. In addition, formation of tetracycline intermediates immediately after the addition of NaYF_4_:Yb,Tm@TiO_2_-Acac core-shell structures, making them a promising material for water purification through the synergy of catalytic and photocatalytic processes.

## Data Availability

The data underlying this article will be available on reasonable request to corresponding authors.

## Supplementary Materials

The following supporting information can be downloaded at: https://www.mdpi.com/article/10.3390/ijms24119441/s1.
