# Supplementary material for: Tetracycline Removal through the Synergy of Catalysis and Photocatalysis by Novel NaYF4:Yb,Tm@TiO2-Acetylacetone Hybrid Core-Shell Structures
Source: Int J Mol Sci. 2023 May 29;24(11):9441. doi: 10.3390/ijms24119441 (PMC10253680; doi:10.3390/ijms24119441)
Supplement: Supplementary file 1 [file ijms-24-09441-s001.zip › ijms-2407821-supplementary.pdf]

Supplementary file:

Tetracycline Removal through the Synergy of Catalysis and Photocatalysis by Novel $NaYF_4$:Yb,Tm@TiO_2-Acetylacetone Hybrid Core-Shell Structures

Lidija Mančić [1,*], Lucas A. Almeida [2], Tamires M. Machado [2], Jessica Gil-Londoño [2], Ivana Dinić [1], Miloš Tomić [1], Smilja Marković [1], Paula Jardim [3] and Bojan A. Marinkovic [2,*]

[1] Institute of Technical Sciences of SASA, 11000 Belgrade, Serbia; ivana.dinic@itn.sanu.ac.rs (I.D.); milos.tomic@itn.sanu.ac.rs (M.T.); smilja.markovic@itn.sanu.ac.rs (S.M.)

[2] Department of Chemical and Materials Engineering, Pontifical Catholic University of Rio de Janeiro (PUC-Rio), Rio de Janeiro 22453-900, Brazil

[3] Department of Metallurgical and Materials Engineering, Federal University of Rio de Janeiro, Rio de Janeiro 21941-853, Brazil

* Correspondence: lidija.mancic@itn.sanu.ac.rs (L.M.); bojan@puc-rio.br (B.A.M.)

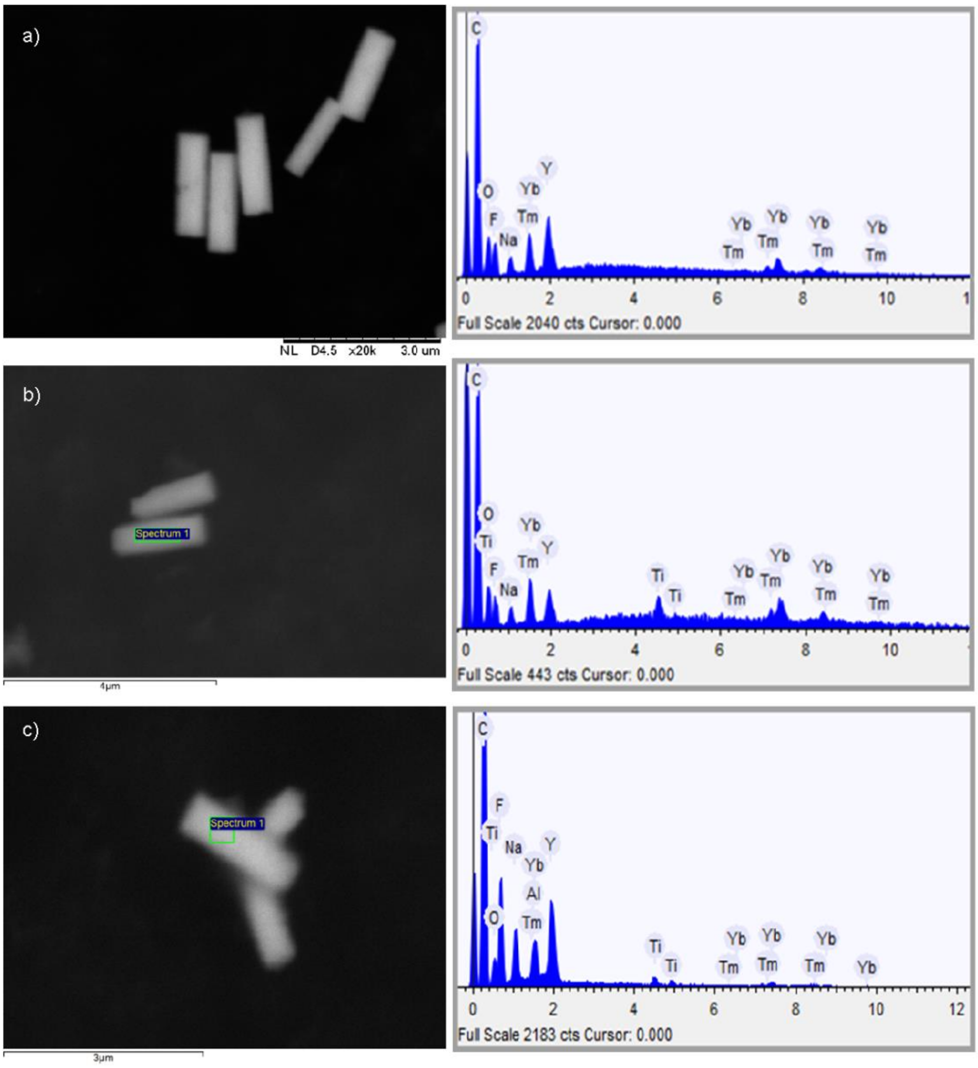

Figure S1. SEM/EDS of $NaYF_4$:Yb,Tm (a) $NaYF_4$:Yb,Tm@TiO_2-Acac (b) and $NaYF_4$:Yb,Tm@TiO_2-Acac 300 core-shell particles (c).

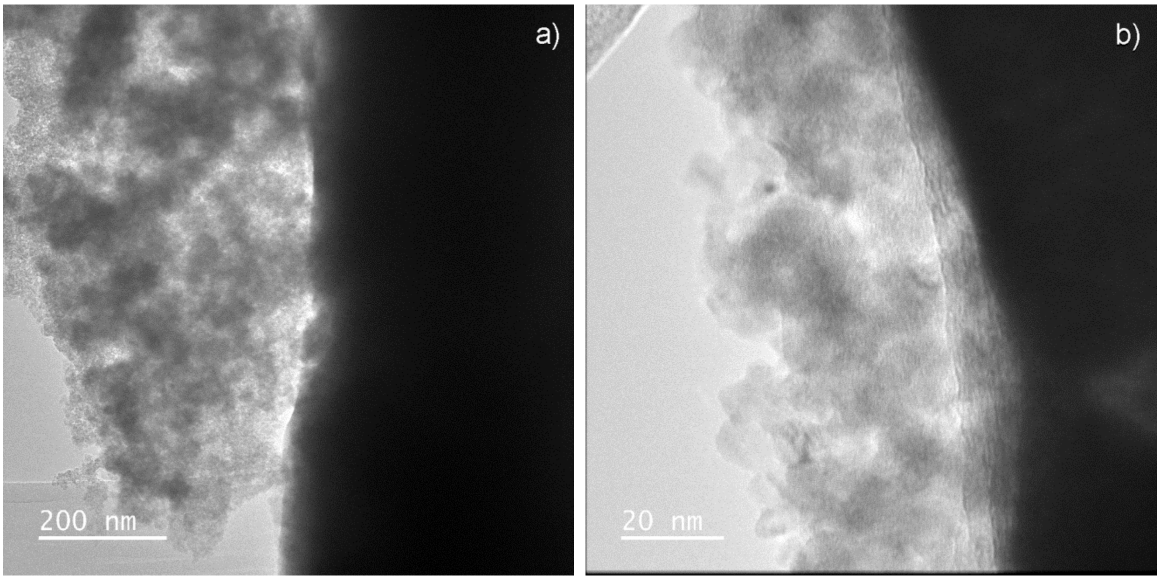

Figure S2. TEM of $NaYF_4$:Yb,Tm@TiO_2-Acac (a) and $NaYF_4$:Yb,Tm@TiO_2-Acac 300 core-shell particles (b).

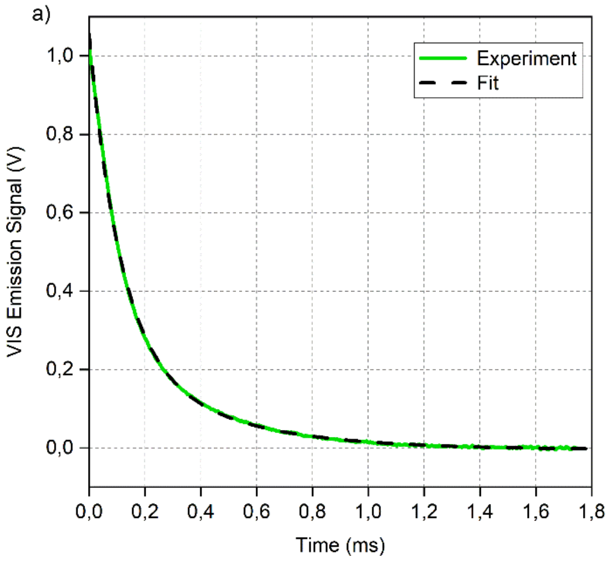

Model	ExpDecay2
Equation	y = y0 + A1*exp(-(x-x0)/t1) + A2*exp(-(x-x0)/t2)
y0	-0,005 ± 4,16396E-5
x0	0,00775 ± --
A1	0,73861 ± --
t1	0,11078 ± 8,33616E-5
A2	0,26332 ± --
t2	0,38832 ± 7,17557E-4
Reduced Chi-	1,46293E-5
R-Square (CO	0,9996
Adj. R-Square	0,9996

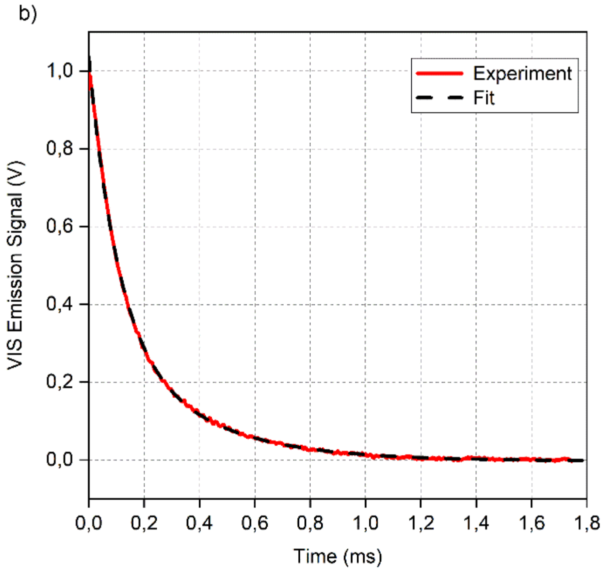

Model	ExpDecay2
Equation	y = y0 + A1*exp(-(x-x0)/t1) + A2*exp(-(x-x0)/t2)
y0	-0,00305 ± 2,93233E-5
x0	0,00294 ± --
A1	0,66558 ± --
t1	0,10287 ± 8,91596E-5
A2	0,35112 ± --
t2	0,32915 ± 4,27944E-4
Reduced Chi-S	1,19352E-5
R-Square (CO	0,99966
Adj. R-Square	0,99966

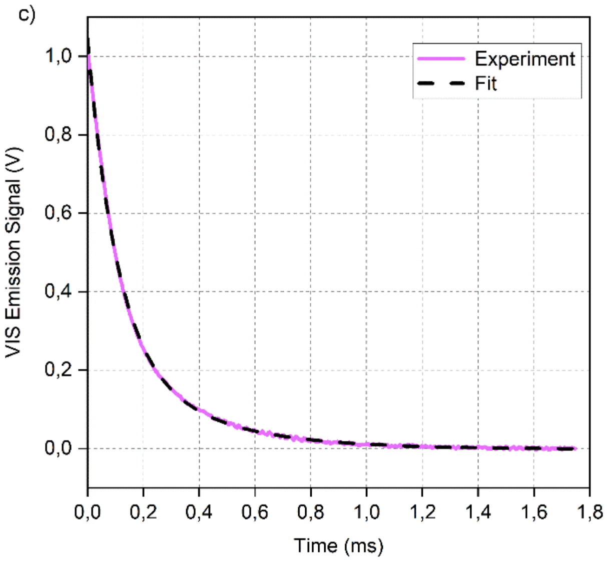

Model	ExpDecay2
Equation	y = y0 + A1*exp(-(x-x0)/t1) + A2*exp(-(x-x0)/t2)
y0	-0,00226 ± 3,45539E-5
x0	0,00685 ± --
A1	0,7303 ± --
t1	0,10406 ± 8,72769E-5
A2	0,26249 ± --
t2	0,33251 ± 6,4589E-4
Reduced Chi-S	1,40026E-5
R-Square (CO	0,99959
Adj. R-Square	0,99959

Figure S3. Decay curves of Vis emission for $NaYF_4$:Yb,Tm particles (a), $NaYF_4$Yb,Tm@TiO_2-Acac (b) and $NaYF_4$Yb,Tm@TiO_2-Acac 300 core-shell structure (c).
